# Unveiling the Ongoing Expansion of Bullheads of the Genus *Ameiurus* (Actinopteri: Siluriformes) in the Dnipro River Basin, Ukraine: New Distributional Records and Genetic Diversity

**DOI:** 10.1002/ece3.74564

**Published:** 2026-11-01

**Authors:** Olena Bielikova, Fedor Čiampor, Oleksandr Didenko, Ladislav Pekárik, Yuliia Kutsokon

**Affiliations:** ^1^ Institute of Fisheries of the National Academy of Agrarian Sciences of Ukraine Kyiv Ukraine; ^2^ Plant Science and Biodiversity Centre Slovak Academy of Sciences Bratislava Slovakia; ^3^ Schmalhausen Institute of Zoology National Academy of Sciences of Ukraine Kyiv Ukraine

**Keywords:** biological invasion, COI, DNA barcoding, freshwater fish, genetic diversity, Ictaluridae, mtDNA

## Abstract

Invasive bullheads of the genus Ameiurus are actively expanding across Eastern Europe, yet accurate species identification remains problematic owing to phenotypic plasticity and high intraspecific morphological variability. This study aims to resolve the taxonomic identity, spatial distribution, and invasion dynamics of Ameiurus populations within the Dnipro River catchment, Ukraine, where mapping the current frontiers of these non‐native species is critical for freshwater conservation. We employed an integrated framework combining laboratory molecular analysis (mtDNA COI barcoding) with field‐based morphological scoring via Discriminant Analysis of Principal Components (DAPC). Sampling was conducted across multiple riverine networks within the catchment to evaluate whether range expansion is driven by natural downstream dispersal or human‐mediated pathways. A total of 66 specimens from six sampling localities were analysed, yielding 66 COI sequences. Phylogenetic reconstruction consistently recovered two discrete, high‐support clades corresponding to *A. nebulosus* and *A. melas* , a topology consistently supported across multiple distance‐based and phylogeny‐based delimitation methods. These results document the continued range expansion of both species in the Dnipro River basin, including the first molecularly confirmed occurrence record of *A. melas* in this basin, previously known only from the Southern Buh and Danube basins. Genetic analysis revealed five COI haplotypes per species, most of which are new to Ukraine, a pattern consistent with multiple independent introduction events or the retention of standing genetic variation from diverse source populations. DAPC profiling identified body colouration, pectoral spine serration, anal fin membrane pigmentation, and gill raker count as the most robust diagnostic traits for reliable field delimitation. The observed lack of isolation‐by‐distance and the allopatric establishment of both species in adjacent water bodies support a ‘jumpy’ colonisation pattern driven by unauthorised human‐mediated translocations rather than natural dispersal. This study provides a scalable, integrated framework to resolve taxonomic ambiguities in cryptic or plastic invasive fish populations. Our findings establish an ecological baseline for range forecasting and risk assessment of Ameiurus in European inland waters, underscoring the urgent need for coordinated molecular monitoring at the leading edges of freshwater biological invasions.

## Graphical abstract

This study investigates the rapid expansion, genetic structure, and phenotypic traits of two non‐native ictalurids, *Ameiurus nebulosus* and *Ameiurus melas* , within the under‐researched Dnipro River basin, Ukraine. Integrating mtDNA COI barcoding with morphological DAPC analysis, we provide the first molecularly verified record of *A. melas* in the region, documenting a significant eastward shift in its global invasive frontier. The findings reveal a distinct genetic and phenotypic substructure, supporting an allopatric, human‐mediated ‘jumpy’ colonisation pattern rather than natural downstream dispersal.

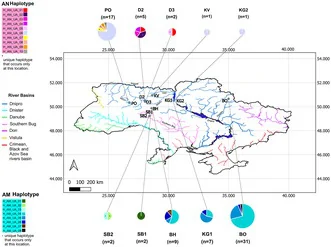

## 1 Introduction

Freshwater ecosystems are among the most severely affected by biological invasions (Kostić et al. 2024), yet they remain disproportionately under‐monitored, particularly at the expanding eastern frontier of European invasion networks. Accurate species identification, effective distribution monitoring, and the development of management strategies are essential for preserving native ecosystems and biodiversity (Comtet et al. 2015; Pergl et al. 2020; Sesay et al. 2024).

North American bullheads of the genus *Ameiurus* Rafinesque, 1820 (Siluriformes: Ictaluridae) represent the invasive fish species that pose a significant threat to European aquatic ecosystems, including those in Ukraine. Specifically, brown bullhead *Ameiurus nebulosus* (Lesueur, 1819) and black bullhead *Ameiurus melas* (Rafinesque, 1820) were introduced to Europe in the 19th century (Kalinowska et al. 2023). Following their introduction, they have been actively spreading across new territories, with recent trends suggesting a rising dominance and potential displacement of brown bullhead by black bullhead in several European regions (Brys et al. 2025). Their success is due to their high ecological plasticity and tolerance to a wide range of environmental conditions, including poor water quality, significant anthropogenic pressure, low dissolved oxygen concentrations, broad temperature ranges and variable pH levels (Ulikowski et al. 2022). This adaptability, encompassing flexible life history strategies, high pollution tolerance, rapid population recovery, developed parental care, and the ability to switch between active predation and carrion consumption depending on habitat complexity and turbidity, collectively facilitates their invasion success (Jaćimović et al. 2023; Preiszner et al. 2024). Numerous studies and reports highlight the substantial negative impact of bullheads on natural aquatic ecosystems, including competition for resources with native species, predation on fish and their eggs, degradation of water quality, and the transmission of parasites and viruses (Aldridge et al. 2017; Magliozzi et al. 2023). Notably, members of the genus *Ameiurus* (especially *A. melas* ) pose a direct threat to Red List taxa, such as the spadefoot toad *Pelobates cultripes* , as documented in Western Europe (Magliozzi et al. 2023). Effective large‐scale control remains challenging due to the lack of a single, fully reliable method (Predojević et al. 2022). Habitat modification and physical removal lack sufficient evidence for sustainable population reduction, while more intensive methods such as rotenone application or mass netting show potential but remain context‐specific (Aldridge et al. 2017; Jaćimović et al. 2023). Natural predation by native species is often insufficient, as predators may prefer alternative prey (Ulikowski et al. 2022; Vanderbush et al. 2022). These findings emphasise the potential for similar negative impacts on native biodiversity in other invaded regions, including Ukraine, where the expansion of bullhead populations could threaten local vulnerable species through analogous ecological pressures. Consequently, monitoring and managing their populations in newly invaded areas, including Ukrainian water bodies, is necessary.

In Ukraine, *A. nebulosus* was first recorded in lakes within the interfluve of the Vistula and Dnipro basins in the late 1930s. Since the early 2010s, it has been consistently reported in the Dnipro basin, particularly in the Prypiat sub‐basin, and has also been found in the Dniester basin (Kutsokon et al. 2018, 2024). In contrast, *A. melas* was previously known only from the Danube basin in Transcarpathia and the Southern Buh basin (Kutsokon et al. 2024; Movchan et al. 2014). However, in the last two years, there has been an increase in new records and ongoing range expansion of the range of *A. melas* in Ukraine. Despite their long presence in Ukraine, detailed data on the distribution, genetic diversity, and ecological impact of *Ameiurus* species remain limited, particularly in the Dnipro River basin, a key invasion hotspot.

Historically, accurate species identification of *Ameiurus*, based on morphological traits, has been problematic due to significant phenotypic plasticity, morphological similarity, and high intraspecific variability (Jaćimović et al. 2023; Nowak et al. 2010). Furthermore, unresolved taxonomic issues, morphological challenges, and potential hybridisation in natural environments complicate their identification (Belle et al. 2021). These factors emphasise the need for accurate molecular methods for species confirmation and delimitation. Analysis of the mitochondrial cytochrome c oxidase subunit I (COI) gene has proven to be a reliable tool for DNA barcoding and phylogenetic studies. The presence of multiple haplotypes in samples also enables the investigation of genetic diversity and compares it with data from other countries. Despite the significant spread of *Ameiurus* in Ukraine and throughout Europe, there are still gaps in our detailed understanding of their current distribution, genetic population structure at new invasion fronts, and effective methods for their distinction. This is particularly relevant for the Dnipro River basin, where new records are actively being documented. Previous records from Ukraine were based primarily on morphological identification, highlighting the need for molecular confirmation.

This study addresses these gaps by documenting the distribution, genetic diversity, and introduction dynamics of both *Ameiurus* species in new localities within the Dnipro River basin, with implications for biodiversity monitoring and management. Specifically, we test whether both species are actively expanding their ranges in the Dnipro basin, examine the genetic evidence for human‐mediated versus natural dispersal, and evaluate the diagnostic value of morphological traits for field identification with direct implications for biodiversity monitoring and management across Eastern Europe.

## 2 Methods

### 2.1 Study Area and Sample Collection

Fish samples were collected from six locations in the Dnipro River basin (Figure 4) during 2023–2024, which represent new records for both *A. nebulosus* and *A. melas* (Figure 1). The sampling sites represent rivers (KV and BH) and ponds (KG1, KG2, BO and PO); detailed information is provided in Table 1.

**FIGURE 1:**
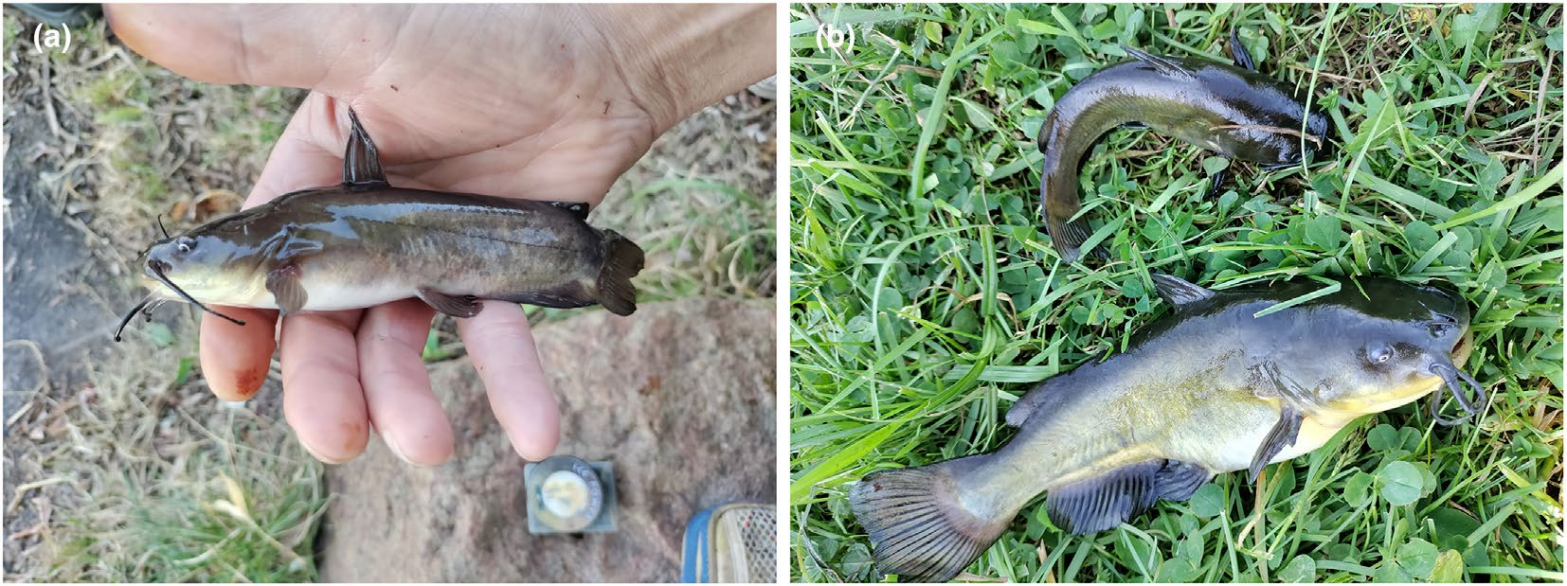
Photographs of bullhead specimens: (a) Brown bullhead ( *Ameiurus nebulosus* ) from Korosten (Uzh River) and (b) Black bullhead ( *Ameiurus melas* ) from Berdychiv (Hnylopiat River). Photographs by Y. Kutsokon and M. Shcherbatiuk, respectively.

**TABLE 1:** Collected samples of bullheads from the Dnipro basin.

ID	Samples	Date		Site	Coordinates		Collector	*n* (*n* for genetic analyses)
BH	A12—A21	2.06	2023	Berdychiv, Hnylopiat River	49.8655	28.58639	Kutsokon Y.	9 (9)
BO	A22—A53	1.04	2024	Boromlia, pond on Boromlia River	50.65963	34.99382	Didenko O., Kutsokon Y.	32 (31)
KG2	A54—A55	17.07	2024	Kyiv, pond 2 in park Kyn‐Grust	50.51957	30.44123	Didenko O., Kutsokon Y., Bekh V.	2 (1)
KG1	A56—A65	17.07	2024	Kyiv, pond 1 in park Kyn‐Grust	50.51978	30.4392	Didenko O., Kutsokon Y., Bekh V.	10 (7)
KV	A66	9.09	2024	Korosten, Uzh river	50.94541	28.64578	Didenko O., Kutsokon Y.	1 (1)
PO	A67—A81	12.09	2024	Polian (ponds)	50.33743	26.74184	Didenko O., Kutsokon Y.	17 (17)

Samples were collected using various methods. At locations BH, KG2, KG1 and PO, fish were captured with a dipnet with specified dimensions (1.5 m handle, 0.5 m net width, 0.7 m net length and 0.5 cm mesh size; maximum capturing depth up to 1 m). At KG2 and KG1, a fishing rod baited with an earthworm was also used. Additionally, specimens were also obtained from local fishermen at KV and BH, and a separate sample was obtained from a pond farm (BO). Specimens obtained from fishermen were part of their legal recreational catch; in these cases, tissue samples were collected from already harvested fish. The chosen combination of dipnetting, angling, and opportunistic sampling was used as the most effective and legally compliant approach for obtaining the required sample size, as electrofishing is not legal in Ukraine. All sample collection procedures complied with ethical guidelines and did not involve invasive animal experiments or harm to the specimens. Fish sampling was conducted as part of the institutional research monitoring programmes of the institutes. As the target species are invasive and not subject to conservation restrictions or specific harvest quotas in Ukraine, no additional collection permits were required for the non‐lethal sampling performed in these public water bodies. Tissue samples (fin clips) were collected from live specimens, which were subsequently released at the site of capture, except for those already harvested by fishers. The genetic material was acquired lawfully for sequencing for non‐commercial academic research in accordance with the principles of the Nagoya Protocol.

### 2.2 DNA Extraction, PCR Amplification and Sequencing

Tissue samples (fragments of the pectoral or pelvic fin) were collected and preserved in 96% ethanol at −20°C until further processing. Genomic DNA was extracted using the Fast Extract DNA solution kit (Avantor), following the manufacturer's protocol. The resulting DNA solution was then diluted at a 1:10 ratio. DNA concentration was quantified using a Quantus fluorometer (Promega).

For molecular analysis, we used the standard animal barcoding fragment (5′ part of the COI mtDNA gene), which allows for direct comparison with large public databases. The primers, PCR amplification conditions, purification of PCR products, and sequencing, as well as subsequent quality control and preparation of sequences for bioinformatic analysis, were performed according to methods detailed in Kutsokon et al. (2024).

### 2.3 Molecular Data Analysis

#### 2.3.1 Species Identification and Phylogenetic Analysis

Species identification was performed by comparing the obtained COI sequences with reference data in the bold systems database (v4) (Ratnasingham et al. 2024; Ratnasingham and Hebert 2007, 2013). The best‐fit nucleotide evolution model for the COI data was determined using iq‐tree multicore version 2.0.7 (Kalyaanamoorthy et al. 2017; Minh et al. 2020) and the Bayesian Information Criterion (BIC). Phylogenetic reconstruction of species relationships was conducted using two approaches: (1) Maximum Likelihood (ML) method, as implemented in iq‐tree 2.0.7 with 1000 bootstrap replicates for branch support assessment; (2) Bayesian Inference (BI), performed in beast 1.10 (Suchard et al. 2018). We used the HKY + G + I nucleotide substitution model, which was identified as the best‐fit model by the BIC. A Strict Clock model was applied, assuming a constant rate of evolution across all branches of the tree, which is a reasonable assumption for this dataset and timescale. The tree prior was based on the Yule Model, suitable for modelling diversification and lineage dispersal processes, especially in the context of invasive species. Two independent Markov Chain Monte Carlo (MCMC) chains were run for 10,000,000 generations, with sampling every 1000 generations, and the first 25% of generations were discarded as ‘burn‐in’. Chain convergence was checked using tracer 1.7 (Rambaut et al. 2018). All chains achieved an effective sample size (ESS) greater than 200 and were combined using logcombiner 1.10.4. The final tree was summarised using treeannotator 1.10.4. All mentioned programmes are part of the beast 1.10 package. Sequences of the related *Ictalurus punctatus* (Rafinesque 1818) (BOLD FFMBH2032‐14) and *Ameiurus natalis* (yellow bullhead) (BOLD CFF544‐16 and EPAMC583‐20) were used as outgroups for correct tree rooting. The Tree Visualisation By One Table (tvbot) web tool (Xie et al. 2023) was used for the visual representation of the phylogenetic tree and the integration of species delimitation data.

#### 2.3.2 Species Delimitation

Several complementary approaches, which increased the reliability of molecular species delimitation and accounted for various aspects of evolutionary processes, were used to define molecular operational taxonomic units (MOTUs) and to delimit species. The methods applied included both distance‐based and phylogeny‐based methods. The distance‐based methods included the Barcode Index Number (BIN) system (Ratnasingham and Hebert 2013), the barcode‐gap approach using the Automatic Barcode Gap Discovery (abgd) software (Puillandre et al. 2012), and the Assemble Species by Automatic Partitioning (asap) method (Puillandre et al. 2021). Both abgd and asap analyses were performed on the spar‐texplorer website https://spartexplorer.mnhn.fr/ (Miralles et al. 2022) using the standard K2P (Kimura 2‐parameter) distance correction.

For phylogeny‐based methods, we used several approaches. On the Maximum Likelihood (ML) phylogenetic tree, we applied the Poisson Tree Processes (PTP) methods, including multi‐rate PTP (mptp) (Kapli et al. 2017). The analysis was conducted using mptp v.0.2.4 software (available on GitHub: https://github.com/Pas‐Kapli/mptp) with the options ‐‐ml (for ML estimation), ‐‐multi (for modelling multiple substitution rates) and ‐‐pvalue 0.001 as the species boundary threshold. We also used mPTP‐MCMC (multi‐rate Poisson Tree Processes MCMC) with a chain length of 50,000,000 generations (‐‐mcmc 50,000,000), sampling every 100,000 generations (‐‐mcmc_sample 100,000) and a burn‐in of 1000,000 generations (‐‐mcmc_burnin 1,000,000). This analysis also included the options ‐‐multi and ‐*p* value 0.001 (with a posterior probability threshold PP ≥ 0.95). For the Bayesian‐built phylogenetic tree, we used the Bayesian Poisson Tree Processes (bPTP) method, available on the bPTP online server (https://species.h‐its.org/) (Zhang et al. 2013). Furthermore, we applied the Yule‐coalescent (GMYC) model‐based approach, which includes both single‐threshold (sGMYC) and multi‐threshold (mGMYC) models for cluster detection (Michonneau 2017). This analysis was performed using the r v4.4.3 environment (R Core Team 2025) and the packages ‘ape’, ‘paran’, ‘rncl’ and ‘splits’ (Dinno 2025; Ezard et al. 2021; Michonneau et al. 2023; Paradis and Schliep 2019).

Although *A. nebulosus* and *A. melas* are morphologically and taxonomically well‐known species, both share the same Barcode Index Number (BOLD:AAA7255), creating ambiguity in automated identification pipelines. Furthermore, the two species show shallow interspecific divergence that falls within the range where single‐threshold approaches are known to fail (Puillandre et al. 2012). Application of multiple complementary algorithms, spanning both distance‐based (ABGD, ASAP and BIN) and phylogeny‐based (mPTP, bPTP and GMYC) approaches, was therefore necessary to rigorously demonstrate that the genetic clusters recovered correspond to species‐level boundaries rather than intraspecific haplotype groups, and to test whether delimitation outcomes were method‐dependent.

#### 2.3.3 Phylogeographic and Genetic Diversity Analysis

Haplotype determination and genetic diversity indices, including number of variable sites (S), number of mutations, G + C content, number of haplotypes (*h*), haplotype diversity (*Hd*), nucleotide diversity (*Pi*), average number of nucleotide differences (*k*), and Theta, were calculated using the dnasp 5.10.01 software (Librado and Rozas 2009). The neutrality tests (Tajima's *D*, Fu and Li's *D* test, Fu and Li's *F* test, Fu's Fs statistic and Strobeck's S statistic) were also conducted in dnasp to detect deviations from the neutral evolution model, which may indicate demographic changes or natural selection. A median‐joining (MJN) haplotype network was constructed using popart 1.7 software (Bandelt et al. 1999; Leigh and Bryant 2015) for visualisation.

Genetic distances between haplotypes and between locations were calculated using the Jukes‐Cantor (JC + G + I) model, chosen based on the highest BIC values for the dataset without outgroups. The corresponding heatmap and tree between locations were constructed in the r environment. The matrix of genetic distances between locations was converted to a dist class object using the ‘ape’ package (Paradis and Schliep 2019). The phylogenetic tree was built using the Neighbour‐Joining (NJ) method, also implemented in the ‘ape’ package. The tree was rooted using the ‘midpoint rooting’ method from the ‘phangorn’ package (Schliep et al. 2017). Tree visualisation was performed using the ‘ggtree’ package (Yu et al. 2017), which is an extension of the ‘ggplot2’ graphics system (Wickham 2016). A heatmap of pairwise genetic distances between locations was also created with the ‘ggplot2’ package.

A map of haplotype distribution by collection site was created using qgis 3.34.11 software. The administrative boundaries of Ukraine were obtained from the GADM (Global Administrative Areas) database, version 4.1 (https://gadm.org/). River basin data (HydroBASINS) and the hydrographic network (HydroRIVERS) were sourced from the HydroSHEDS project, provided by the World Wildlife Fund (WWF) and Bernhard Lehner, McGill University (https://www.hydrosheds.org/).

The relationship between genetic and geographic distances was assessed qualitatively by examining pairwise Jukes‐Cantor distances among sampling localities in the context of their geographic positions and hydrological connectivity. A formal isolation‐by‐distance test (Mantel test) was not conducted, as the limited sample sizes at several localities (KG2: *n* = 1; KV: *n* = 1; SB1: *n* = 2; SB2: *n* = 2) and the presence of localities in hydrologically isolated drainage basins precluded a statistically meaningful and biologically interpretable analysis.

### 2.4 Morphological Analysis

Statistical analysis was conducted to assess the potential for species delimitation based on morphological characteristics. The analysed traits (Table S2) included meristic (count) and qualitative (descriptive) characters significant for differentiating the two species. Meristic characters included the total number of anal fin rays (A), gill raker count (Sp.br.), caudal fin rays count (C), and dorsal fin branched rays count (D). Qualitative characters were converted into ordinal scales to reflect the biological gradient from 0 (no or low development) to 1 (fully developed) of trait development, or were expressed as presence/absence of the specific trait: anal fin base enlargement (BE) as 0—weakly developed, 1—well developed; anal fin membrane pigmentation (MB) as 0—light, 0.5—grey, 1—dark; body colouration pattern (BC) as 0—no spots, 0.5—bit spotted, 1—fully spotted; development of serration on pectoral‐fin spine (SE) as 0—small, 0.5—medium, 1—well developed; pectoral fin serration near tip 0—absent, 1—present.

Discriminant Analysis of Principal Components (DAPC) was applied to morphological trait data to assess species separation, using molecularly confirmed species identity as the basis for group assignment. One discrimination function was utilised to separate these two species. Data was scaled prior to the analysis to have a mean of 0 and a standard deviation of 1, and thus to avoid the overestimation of data with a high range. To prevent overfitting and evaluate classification accuracy, the optimal number of principal components (PCs) to retain was determined using the optim.a.score cross‐validation function from the adegenet package. The discriminatory contribution of individual morphological traits was evaluated using DAPC variable loadings (var.contr). Posterior membership probabilities were calculated for each individual using an ambiguity probability threshold of 0.95 to evaluate assignment confidence. DAPC results were fitted to a 2D space to demonstrate the successful discrimination of the species. All statistical analyses were performed in the r statistical environment using the ‘adegenet’ package (Jombart 2008) for DAPC implementation and ‘ggplot2’ (Wickham 2016), ‘ggrepel’ (Slowikowski 2024) and ‘gridExtra’ (Auguie and Antonov 2017) packages for visualisation.

## 3 Results

### 3.1 Phylogenetic Analysis and Species Delimitation

In total, 66 bullhead (*Ameiurus*) specimens were successfully sequenced, yielding a 609 bp COI fragment. Eighteen samples from three locations (KG2, PO, KV) were identified in the BOLD database as *A. nebulosus* , while the remaining 48 samples from three other locations (BH, BO, KG1) were identified as *A. melas* . The molecular identifications were fully consistent with the initial morphological determinations.

Bayesian Inference analysis produced a tree that clearly separated individuals of the two species with high confidence (posterior probabilities = 1) (Figure 2). Other species delimitation methods also showed a clear separation of the two species (ASAP, ABGD, mPTP (PTP 0.001), mPTP‐MCMC (PP ≥ 0.95), bPTP and GMYC single threshold), with individuals assigned to their respective species clusters. The multiple‐threshold GMYC analysis subdivided *A. melas* into seven clusters and *A. nebulosus* into five, which were consistent with the branching patterns observed in the phylogenetic tree.

**FIGURE 2:**
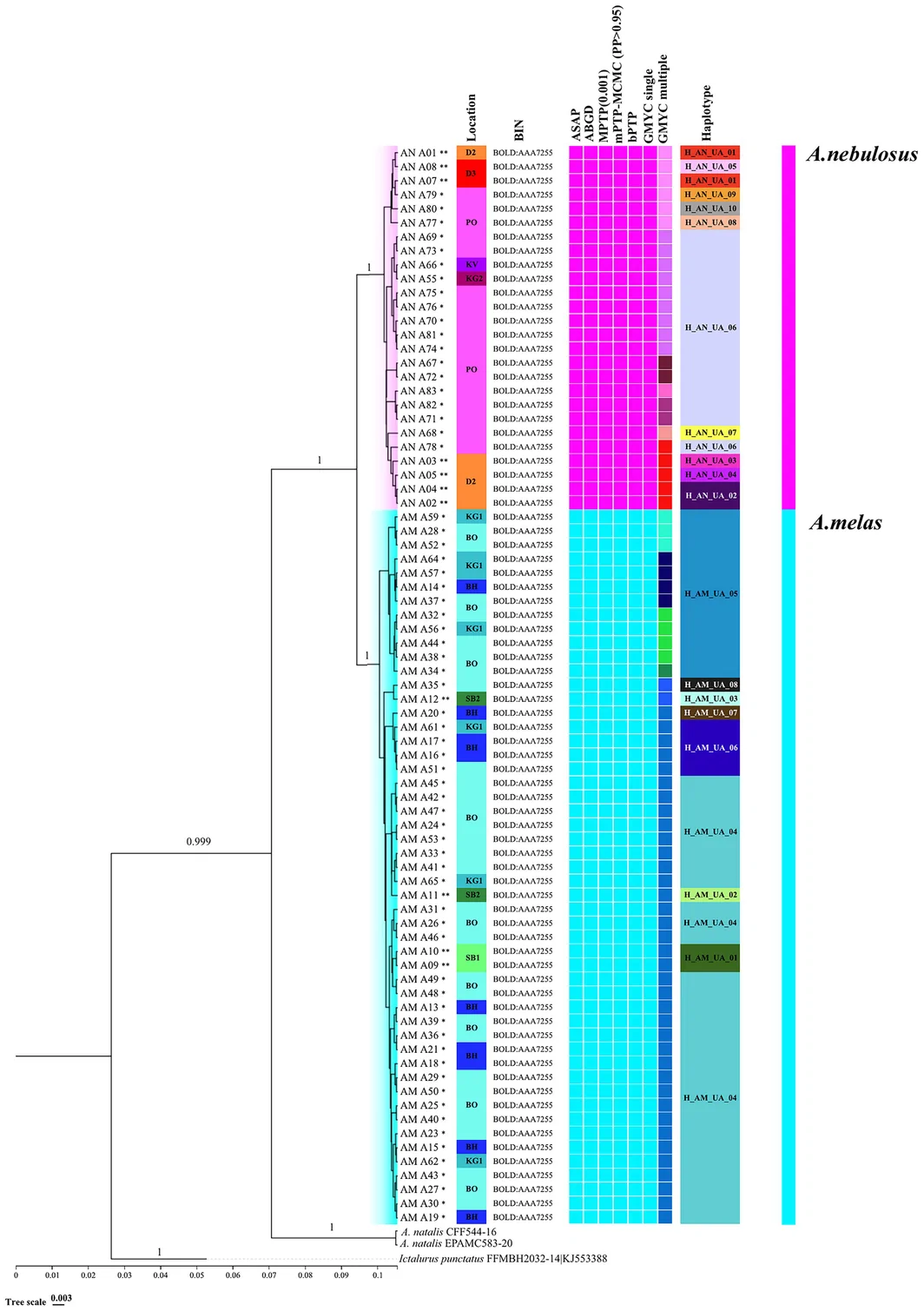
Bayesian phylogenetic reconstruction of the relationship between *A. nebulosus* and *A. melas* in Ukraine using the cytochrome c oxidase I (COI) fragment, including comparison with other species delimitation methods (ASAP, ABGD, mPTP, bPTP, GMYC). Numbers above the nodes denote posterior probabilities. ‘*’ indicates data from the current study, ‘**’ indicates data from Kutsokon et al. (2024).

In the full dataset (AN+AM), 16 variable sites and 18 mutations were detected. Detailed genetic diversity indices for the different populations are provided in Table S1.

### 3.2 Genetic Structure and Diversity

In the 609‐bp long analysed fragment, G + C content was similar between species (0.462–0.463). In *A. melas* , four variable sites and four mutations were found, and in *A. nebulosus* , there were three variable sites and 4 mutations. For samples from new invasion locations in this study, five haplotypes were identified for each species. Haplotype diversity (Hd) was 0.558 for *A. melas* and 0.386 for *A. nebulosus* ; nucleotide diversity (Pi) was 0.001 for both species. Neutrality tests yielded non‐significant results for both species.

The positive Tajima's *D* value for the combined AN+AM group was 1.683, but it was statistically non‐significant (*p* > 0.10). The lack of significance for Fu and Li's *D* and *F* test statistics indicates no deviation from the neutral model. Fu's Fs had negative values (−2.203 for *A. nebulosus* , −1.181 for *A. melas* ), but they were not statistically significant (*p* > 0.10), except Strobeck's S for the combined AN+AM sample (0.055, *p* = 0.031).

The haplotype network analysis, constructed using both our new and previously collected samples from Ukraine (Figure 3), clearly distinguished two major haplotype clusters separated by 12 mutational steps. Within each species cluster, a branched structure with both common and rare haplotypes was observed. The haplotypes identified in this study were not previously recorded in Ukraine based on previous reports (e.g., Kutsokon et al. 2024; locations D2, D3, SB1 and SB2). A comparison with publicly available sequences from BOLD showed that H_AM_UA_04 was identical to the most prevalent *A. melas* haplotype found in these databases, and H_AN_UA_06 was similar to the most common *A. nebulosus* haplotype. All other haplotypes we discovered in this work are new. The *A. melas* haplotypes showed more mutational steps within the network compared to *A. nebulosus* .

**FIGURE 3:**
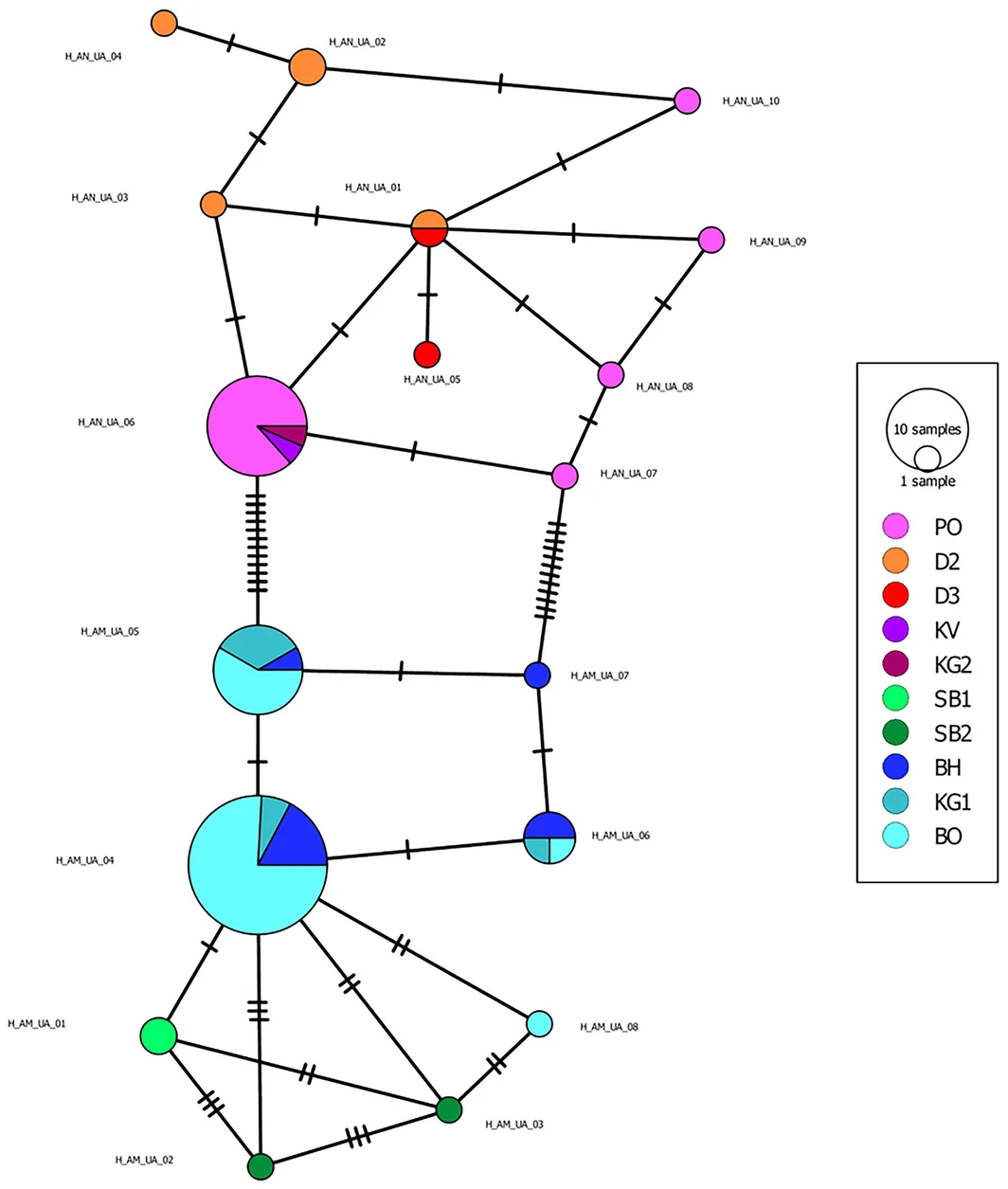
Median‐joining (MJN) haplotype network for *Ameiurus* species in Ukraine based on *COI* sequences (using haplotypes from previous studies in Ukraine: locations D2, D3, SB1 and SB2). Haplotypes starting with ‘H_AN_UA’ correspond to *A. nebulosus* , and those starting with ‘H_AM_UA’ correspond to *A. melas*.

Genetic distances between haplotypes were calculated based on the Jukes‐Cantor model. Intraspecific distances ranged from 0.00168 to 0.00721 for *A. nebulosus* and from 0.00168 to 0.00923 for *A. melas* . The distances between the two species are significantly higher, ranging from 0.02637 to 0.0463. The heatmap (Figure S1) shows clear differences between haplotypes of the two species.

Genetic distances between populations confirmed a clear differentiation between the species (Figure S2). Within *A. nebulosus* , the KV and KG2 populations were not differentiated, as the same single haplotype was found at both locations. The PO population was equidistant from these two populations (distance 0.00071). Regarding *A. melas* , the BH population was most genetically distant from KG1 (0.00156). The BO population was equidistant from BH and KG1 (distance 0.0013).

All newly documented invasion localities are situated within the Dnipro River basin (Figure 4). The two species were not found to co‐occur at any of the sampled localities; each of the two adjacent ponds (KG1 and KG2) contained exclusively one species.

**FIGURE 4:**
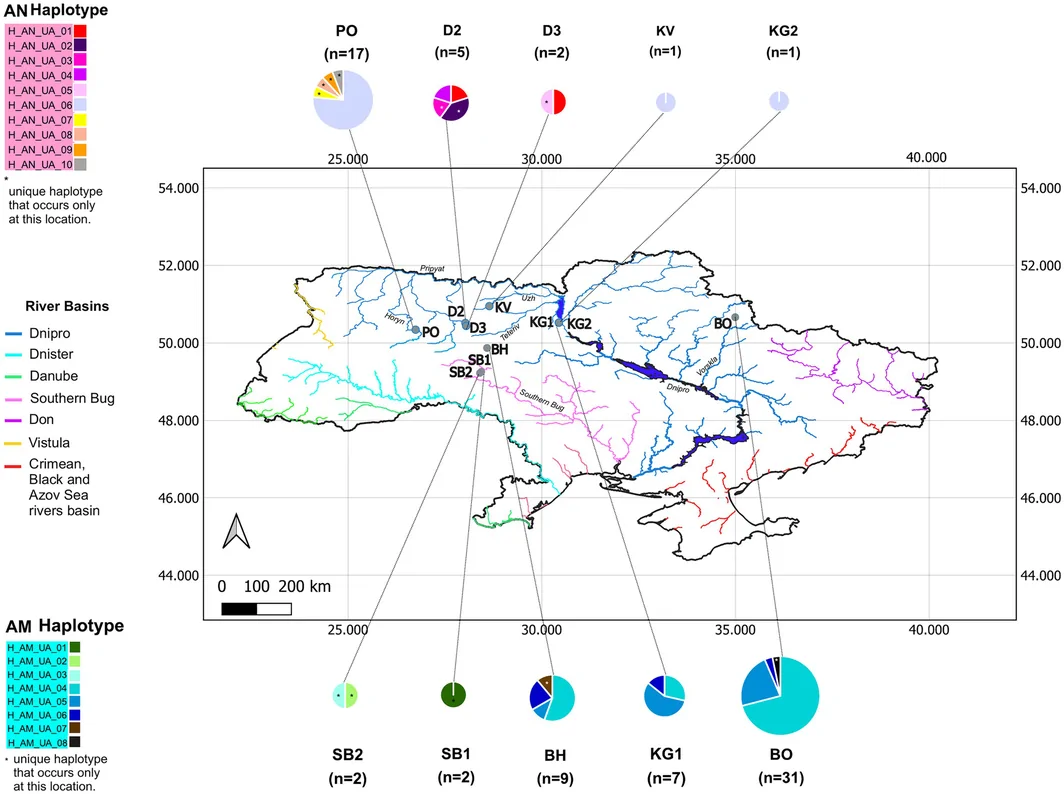
Distribution of *A. nebulosus* (AN) and *A. melas* (AM) haplotypes by collection site (using haplotypes from previous studies in Ukraine: locations D2, D3, SB1 and SB2). Site codes correspond to Table 1. The map was generated using QGIS v.3.34.11; administrative boundaries from GADM v.4.1; hydrographic data from HydroSHEDS. Map projection: WGS 84.

The *A. nebulosus* haplotypes from the new Dnipro basin sites were identical to each other but did not match haplotypes from previous studies (locations D2 and D3). The *A. melas* haplotypes in the Dnipro basin were similar to each other but were distinct from the haplotypes from the Southern Buh basin found in previous studies.

### 3.3 Morphological Analysis

Morphological analysis was conducted in parallel to assess the discriminatory power of selected morphological traits for species‐level identification. Optimization via optim.a.score indicated that retaining one PC was optimal. The PCA biplot of the DAPC results on scaled data demonstrated a clear and robust separation between *A. melas* and *A. nebulosus* along the single discriminant function (Figure 5). Group centroids were distinctly separated, and individual points clustered tightly within their respective species' regions, confirming strong morphological differentiation. Evaluation of variable loadings under scaled data revealed that species separation was driven by four primary traits: development of serrations on the pectoral‐fin spine (contribution of 0.232), body colouration pattern (0.229), anal fin membrane black pigmentation (0.186) and gill raker count (0.165). Morphologically, *A. nebulosus* showed a more spotted body pattern, well‐developed serration on the pectoral fin, including the presence of serration on the tip, while *A. melas* showed higher gill raker counts and darker anal fin membrane pigmentation (Figure S3). The mean posterior probabilities for the group assignment indicate a high confidence in the assignment of individuals to the species based on morphological traits; *A. melas* with a mean of 1.0 (*N* = 42, SD = 0.0001) and *A. nebulosus* with a mean of 0.9996 (*N* = 19, SD = 0.0017). Under alternative scaling (unscaled raw data), one individual (KG2_2) fell below the 0.95 assignment threshold; this likely reflects the very small sample size at this locality (*n* = 1) rather than genuine morphological ambiguity.

**FIGURE 5:**
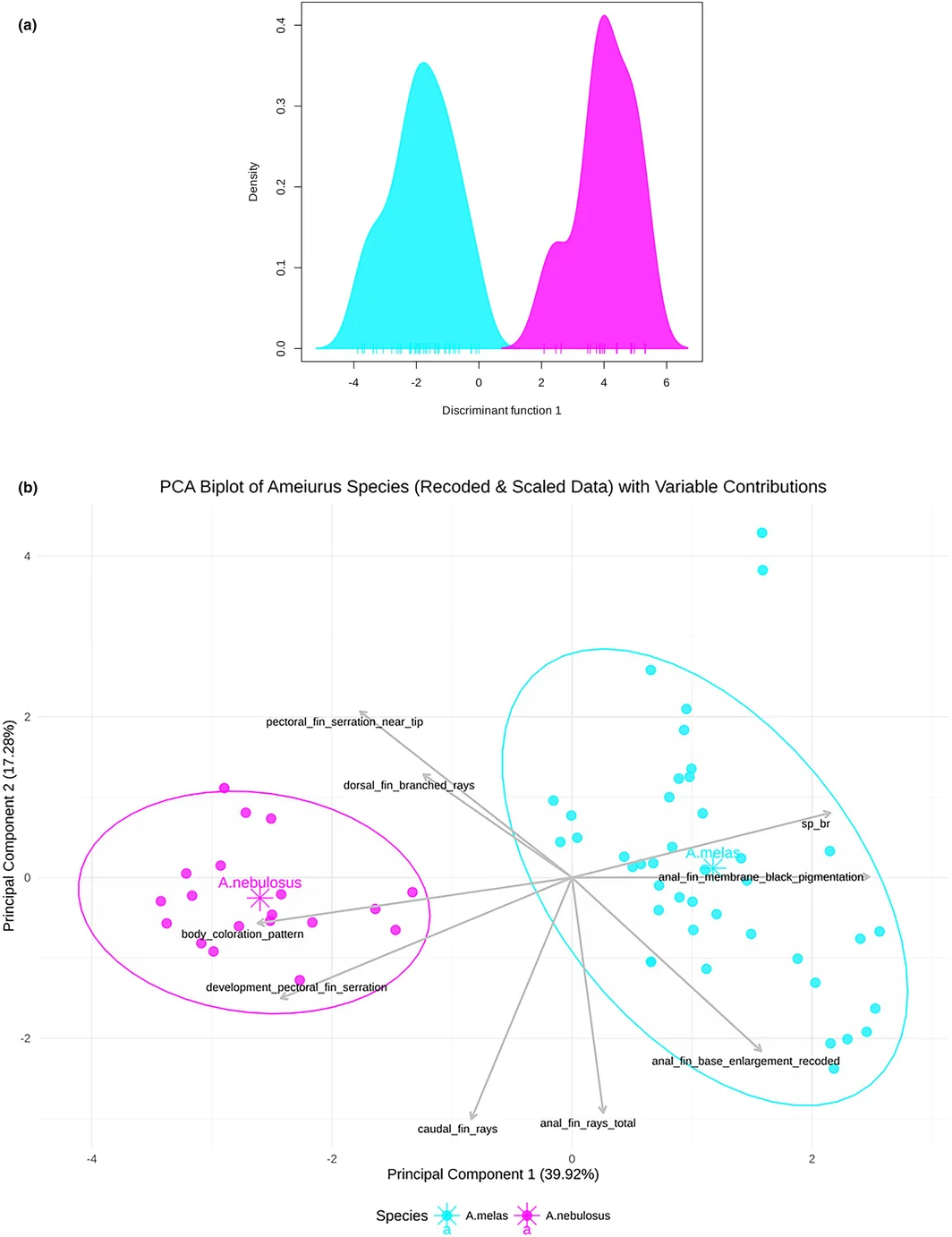
Morphological Analysis: (a) PCA biplot of the Discriminant Analysis of Principal Components, with clear separation of both species and strength and direction of morphological traits. (b) DAPC Scatter Plot with Variable Contributions that visualises the individual samples and their relationships along the discriminant function.

## 4 Discussion

Invasive and non‐native species pose one of the most significant threats to the preservation of native biodiversity and the functioning of ecosystems; freshwater ecosystems are arguably the most vulnerable and, at the same time, the most affected in this regard. This study aimed to better understand the population status of non‐native catfish in Ukraine. By integrating molecular and morphological evidence, this study provides a comprehensive picture of the current distribution of invasive *Ameiurus* bullheads in the Dnipro River basin in Ukraine, addressing species delimitation, genetic diversity, and spatial distribution patterns, along with the likely introduction mechanisms and their implications for monitoring and management.

### 4.1 Species Delimitation and Identification Methods

Genetic and morphological analyses clearly confirmed the presence of two distinct groups ( *A. nebulosus* and *A. melas* ) in new localities within the Dnipro River basin, Ukraine. The mtDNA marker, analysed using multiple species delimitation algorithms, consistently assigned all sampled individuals to discrete monophyletic clades. The absence of haplotype mixing between groups is consistent with the genetic differentiation expected of two distinct biological species, although both have been assigned the same Barcode Index Number (BOLD:AAA7255). The existence of two distinct species is also supported by a recent multilocus phylogenetic study (Janzen et al. 2023). Therefore, this discrepancy likely reflects the clustering thresholds of the BIN algorithm, which may not be optimal in all cases, rather than actual ambiguity in species status. We note that mitochondrial markers alone cannot detect nuclear introgression or ongoing hybridisation. Although this possibility has been documented in both native and invaded ranges (Walter et al. 2014; Béres et al. 2017), it cannot be formally excluded based on the present dataset. However, the absence of sympatric co‐occurrence at any sampled locality reduces the likelihood of recent hybridisation in the studied populations.

In addition to molecular delimitation, DAPC of morphological traits identified four characters as the primary contributors to species separation (pectoral‐fin spine serration, body colouration pattern, anal fin membrane pigmentation and gill raker count), well supported by high discriminatory power. These results are consistent with the diagnostic framework of Rutkayová et al. (2013), who reported 96.4% classification accuracy using discriminant analysis in the Central European dataset, and extend their applicability to a newly invaded Eastern European range. Mean posterior probabilities of group assignment were high for both *A. melas* (1.000 ± 0.0001) and *A. nebulosus* (0.9996 ± 0.0017), confirming that this trait combination provides reliable species‐level identification for adult individuals under field conditions. Conversely, total anal fin ray count and caudal fin ray count contributed negligibly to species separation in the present dataset, suggesting that their diagnostic value may be population‐specific or confounded by age‐related allometric variation, as documented by Ulikowski et al. (2022). While morphology‐based identification remains practical for adult specimens of typical condition, COI barcoding provides unambiguous resolution across all life stages and preservation states, effectively bypassing the constraints imposed by phenotypic plasticity at active invasion fronts. The integration of both approaches, as demonstrated here, offers a scalable and reproducible framework for the early detection and ongoing monitoring of *Ameiurus* across the broader Eastern European invasion range.

### 4.2 Genetic Diversity and Introduction History

Examination of the genetic diversity revealed new haplotypes not previously recorded in Ukrainian populations, indicating that the genetic pool of both species in the Dnipro basin is broader than documented to date (Kutsokon et al. 2024). The multiple‐threshold GMYC analysis further suggested distinct substructuring within each species, consistent with a complex introduction history. The low nucleotide diversity (Pi) recorded for both species is consistent with reduced genetic variation typically observed in newly established invasive populations (Grapputo et al. 2006; Li et al. 2022; Sahoo et al. 2020; Tkachenko et al. 2025). This pattern may reflect a founder effect during the initial stages of introduction, а demographic bottleneck, or simply the relatively recent arrival of the species in the study area. However, given that this study focuses on documenting new invasion localities, and that stochastic small‐scale introductions cannot be excluded, the present mitochondrial dataset provides only a preliminary view of population genetic structure, and finer resolution of invasion dynamics will require broader geographic sampling and nuclear genomic profiling.

The two species showed contrasting patterns of haplotype diversity (Hd) that warrant separate interpretation. In *A. melas* , Hd substantially exceeds values calculated from available COI sequences for other European populations (BOLD, 25 March 2026) despite a similarly low Pi. Such a combination may suggest the introduction of individuals from multiple genetically distinct source populations. However, alternative explanations, such as standing genetic variation in a single diverse source population or secondary spread from already established invasive populations elsewhere in Europe, cannot be excluded based on mitochondrial data alone. Notably, available sequences for *A. melas* from its native North American range show extremely low diversity in public databases, which most likely reflects limited geographic coverage of reference sequences rather than the true state of native populations. The pattern observed here differs from earlier findings for *A. melas* in Ukraine that indicated lower diversity (Kutsokon et al. 2024). This may suggest that introductions from new sources are ongoing, although the dataset remains insufficient for definitive conclusions about introduction dynamics.

For *A. nebulosus* , both Hd and Pi were lower in our dataset than in its native range and other European invaded populations (BOLD, 25 March 2026), suggesting a more constrained introduction history in Ukraine. Whether this reflects fewer source populations, a stronger initial bottleneck, or a later arrival in the study area cannot be determined from the present data. Non‐significant neutrality test results for both species did not provide unambiguous evidence of recent demographic expansion, which is not unexpected given the relatively short timeframe since colonisation.

Qualitative examination of pairwise genetic distances revealed remarkable sequence similarity within each *Ameiurus* lineage across Ukraine, with Jukes‐Cantor distances not exceeding 0.0057 for *A. melas* and 0.0037 for *A. nebulosus* . No positive relationship between genetic and geographic distances was observed along river corridors. Paradoxically, the geographically distant *A. melas* populations of BH (Zhytomyr region) and BO (Sumy region), separated by over 400 km within the Dnipro basin, exhibited the lowest pairwise genetic distance across all locality pairs (*d* = 0.00129). Furthermore, *A. melas* populations from the Dnipro catchment shared near‐identical haplotypes (*d* ≤ 0.0057) with those from the hydrologically isolated Southern Buh basin (SB1, SB2). Because no continuous freshwater route connects these two drainage systems, natural in‐stream dispersal cannot physically account for this spatial pattern. Considered alongside the known history of unauthorised fish transfers in the region, these biogeographical patterns suggest that human‐mediated translocations represent an important driver of range expansion, though we acknowledge that genetic drift in small, isolated populations could also contribute to the observed genetic discontinuities, and that broader sampling would be needed to resolve these processes definitively. For *A. nebulosus* , a more dualistic pattern is apparent: the near‐continuous distribution in the Prypiat sub‐basin may reflect natural eastward spread, whereas fragmented records elsewhere in the Dnipro catchment are more consistent with independent translocation events. The genetic similarity among *A. nebulosus* localities within the Dnipro basin (*d* ≤ 0.00071 between KG2, KV and PO) contrasts with substantially higher divergence from Dniester basin populations (D2, D3; *d* = 0.00256–0.00367), further supporting the interpretation that Dnipro basin populations represent a discrete introduction event rather than natural range expansion from previously established populations.

This study presents the first molecularly confirmed occurrence record of *A. melas* in the Dnipro River basin, substantially extending its documented range in Ukraine beyond the Danube basin in Transcarpathia (Movchan et al. 2014) and the Southern Buh basin (Kutsokon et al. 2024). We emphasise that this constitutes simultaneously the first occurrence record and the first molecularly confirmed occurrence record for this basin, as *A. melas* had not previously been reported from the Dnipro basin in any published literature, either morphologically or molecularly. Support for the novelty of these records comes from prior systematic ichthyological surveys conducted at or near several of the newly colonised localities, including the Hnylopiat River near Berdychiv (Kutsokon and Roman 2018) and the Boromlia River pond system (Kutsokon et al. 2022), in which Ameiurus was not detected. These publications indicate that the species was absent from these specific sites in the recent past, reducing the likelihood that current records merely reflect previously undetected populations. Additionally, information obtained from local recreational fishers at the newly colonised sites consistently indicated that both species had first been observed approximately 2–3 years prior to our sampling, further supporting the interpretation that these represent genuine recent establishment events. The presence of individuals of varying size classes at the Boromlia locality (BO) is consistent with early population establishment, though multi‐year demographic monitoring would be required to confirm a fully self‐sustaining population. Although *A. nebulosus* was already known from the Dnipro basin, our data document its continued spread and identify new localities. The apparent discontinuities in the distribution of both species should be interpreted with caution, as it remains unclear whether these reflect genuine population fragmentation or sampling gaps due to the lack of systematic survey data across the basin. Records from citizen‐science platforms can provide supplementary occurrence signals, for example, additional *A. nebulosus* observations have been reported in the Southern Buh basin (e.g., at Uman in 2024 and Letychiv in 2025) and the Desna River in 2023 (iNaturalist community 2025; GBIF 2025), but require expert validation before use as distributional evidence. We acknowledge that several sampling localities were represented by only one or two individuals for genetic analysis (KV: *n* = 1; KG2: *n* = 1; BH: *n* = 2), which limits conclusions regarding local haplotype diversity and population structure at these sites. These small sample sizes reflect the early stage of invasion monitoring at newly detected localities, where population densities are typically low and access is logistically constrained. Broader geographic sampling and larger per‐locality sample sizes will be necessary to more fully characterise the genetic architecture of *Ameiurus* populations across the Dnipro basin as populations become more established. A reported record from Letychiv proved upon verification to be a misidentification already propagated to GBIF, illustrating the risks of uncritical reliance on unverified data in range assessments. Future monitoring should therefore integrate environmental DNA (eDNA) analyses, which offer greater sensitivity for early detection, particularly where electrofishing is legally restricted (Bonk et al. 2024; Mruk et al. 2024).

Although both species now occur in the Dnipro basin, they were not found in sympatry during this study. The occurrence of each species exclusively in one of two adjacent, hydraulically connected ponds (KG1 and KG2) is more consistent with separate introduction events than with natural colonisation. The proximity of both species within the same river system raises questions about potential future interactions, including competitive exclusion, a process documented in several European countries where the spread of *A. melas* has coincided with the decline of *A. nebulosus* (Bouros et al. 2016; Copp et al. 2016), and hybridisation, recorded in both native and invaded ranges (Walter et al. 2014; Béres et al. 2017; Brys et al. 2025). Under current conditions, hybridisation appears unlikely given the clear genetic and morphological separation observed here, but continued monitoring is warranted to detect any change in this status.

### 4.3 Conservation Implications

The successful establishment and ongoing expansion of *Ameiurus* species documented here are consistent with the known ecological plasticity of these fish, which enables their persistence across a broad range of environmental conditions, including degraded and heavily modified water bodies (Ulikowski et al. 2022; Preiszner et al. 2024). These observations align with previous studies suggesting that habitat degradation and anthropogenic stressors, particularly hydromorphological alterations and water pollution, may facilitate the establishment of invasive freshwater fish species (Mruk et al. 2024). Whether such mechanisms have contributed to *Ameiurus* establishment in the studied Ukrainian localities cannot be determined from the present data alone, as habitat quality and native community composition were not systematically assessed. The discontinuous, human‐facilitated dispersal pattern identified in the present study reinforces this picture: rather than reflecting passive range expansion, the current distribution of both species in the Dnipro basin appears to result from repeated, unauthorised translocations, a process likely compounded by the socio‐economic instability that hampers systematic monitoring and facilitates unauthorised fish transfers. The ongoing war in Ukraine further increases ecosystem vulnerability to biological invasions while simultaneously restricting field monitoring due to safety risks and restricted access to parts of the country.

Our findings, particularly the occurrence of both species in close proximity within Kyn‐Grust Park, highlight the urgent need to transition from sporadic recording to a coordinated, basin‐wide monitoring strategy. Such a strategy should be designed to detect whether both species will eventually form sympatric populations, whether competitive exclusion will occur and whether hybridisation may develop in ways that would further complicate species identification and management.

Future research should also move beyond distributional data towards a detailed assessment of trophic interactions in Ukrainian water bodies. Available studies from other European invaded systems indicate that both *A. melas* and *A. nebulosus* function as trophically flexible generalists whose diet composition varies substantially among localities depending on habitat characteristics and prey availability (Rechulicz and Plaska 2019; Leunda et al. 2008; Radenkovic et al. 2026). For *A. melas* specifically, stable isotope analyses suggest that predation on native fishes, rather than direct resource competition, may represent the primary mechanism of ecological impact (Czeglédi et al. 2024), while gut‐content studies confirm its capacity for direct predation on native ichthyofauna across diverse European systems (Leunda et al. 2008). Given this inter‐population variability, extrapolation from existing European studies to Ukrainian populations is not straightforward. DNA‐based dietary approaches and stable isotope analyses (Traugott et al. 2021) would therefore allow these patterns to be evaluated specifically for the Dnipro basin. This would help clarify the extent to which *A. melas* and *A. nebulosus* compete with each other or with native taxa, representing questions that remain entirely unresolved for Eastern European river systems. As current data are insufficient to predict the maximum colonisation range of these species or their potential spread into brackish systems, long‐term monitoring should be integrated with ecological modelling and habitat‐suitability assessments (Kim et al. 2025) to support future risk analysis. To achieve finer resolution of population genomic architecture, contemporary gene flow, and introgression patterns, future studies should transition from single‐locus mitochondrial barcoding to the high‐throughput nuclear profiling, specifically targeting microsatellites or SNPs (Gutiérrez‐Barragán et al. 2025; Hunnicutt et al. 2005; Walter et al. 2014). Ultimately, raising public awareness of the risks associated with unauthorised fish transfers remains an essential component of preventing further introductions and protecting native aquatic biodiversity in Ukraine.

### 4.4 Conclusions

This study confirms the continued eastward expansion of *A. nebulosus* and provides the first molecularly verified record of *A. melas* in the Dnipro River basin, though sympatric establishment of the two species was not detected at any of the sampled localities. The complex pattern of genetic diversity, population structure, and the observed disconnect between genetic and geographic distances suggests multiple, anthropogenically mediated introductions rather than natural spread. Furthermore, the verified distributional records and population genetic data presented here establish a baseline for future range forecasting and ecological risk assessment in the Dnipro catchment. These findings inform the development of targeted monitoring and management strategies for these high‐impact invasive taxa in Ukraine.

## Author Contributions

**Olena Bielikova:** formal analysis (equal), investigation (equal), methodology (equal), visualization (equal), writing – original draft (equal), writing – review and editing (equal). **Fedor Čiampor:** formal analysis (equal), investigation (equal), methodology (equal), writing – review and editing (equal). **Oleksandr Didenko:** formal analysis (equal), investigation (equal), writing – review and editing (equal). **Ladislav Pekárik:** formal analysis (equal), investigation (equal), writing – review and editing (equal). **Yuliia Kutsokon:** conceptualization (equal), formal analysis (equal), investigation (equal), methodology (equal), writing – original draft (equal), writing – review and editing (equal).

## Funding

This study was supported by the European Union NextGenerationEU under the ‘Scholarships for excellent researchers threatened by the conflict in Ukraine’ programme, specifically through projects No. 09I03‐03‐V01‐00004 for Kutsokon Yuliia and No. 09I03‐03‐V01‐00075 for Bielikova Olena.

## Ethics Statement

All manipulations with fish samples were conducted following the recommendations of the European Convention for the Protection of Vertebrate Animals Used for Research and Other Scientific Purposes (Strasbourg, 1986) ETS No. 123 and UFRC 2014 (Use of Fishes in Research (2014) Guidelines for the use of fishes in research. American Fisheries Society, Bethesda, Maryland. https://fisheries.org/docs/wp/Guidelines‐for‐Use‐of‐Fishes.pdf). For specimens collected by the research team, sampling was performed using non‐lethal methods; a small portion of the pectoral fin was collected, after which all individuals were immediately released back into their natural habitat at the site of capture. For specimens obtained from local fishers or pond farms, tissue samples were taken from fish already harvested for human consumption or aquaculture purposes. No invasive animal experiments were performed, and no animals were sacrificed specifically for the purposes of this study.

## Conflicts of Interest

The authors declare no conflicts of interest.

## Supporting information


**Table S1:** Summary of Genetic Diversity and Neutrality Tests for *Ameiurus* Species in the Studied Populations.
**Table S2:** Comparison of Morphological Traits and Identification of *A. melas* and *A. nebulosus* in Samples from New Locations.
**Figure S1:** Matrix of pairwise genetic distances (Jukes‐Cantor model) between *Ameiurus* spp. haplotypes, demonstrating species delimitation.
**Figure S2:** Dendrogram of *Ameiurus* population clustering from different localities, constructed using the Neighbour‐Joining method on pairwise genetic distances with midpoint rooting. Differences in colours and distances between clusters reflect the level of genetic differentiation among the studied populations.
**Figure S3:** Distribution of morphological trait values for *Ameiurus melas* and *A. nebulosus* across all sampled individuals. Box plots show the distribution of meristic characters (anal fin rays, gill raker count, caudal fin rays); bar charts show the proportional distribution of qualitative characters (dorsal fin branched rays, anal fin base enlargement, anal fin membrane pigmentation, body colouration pattern, pectoral fin serration development, pectoral fin serration near tip). Trait abbreviations correspond to those defined in Table S2.

## Data Availability

The DNA sequences generated in this study are deposited in GenBank under accession numbers (QB228529–QB228594) and the Barcode of Life Data System (BOLD) under the dataset DOI: 10.5883/DS‐AMEIUA2. Sampling metadata and morphological scoring data are permanently archived and publicly accessible in the Zenodo repository at DOI: https://doi.org/10.5281/zenodo.19467731.
