# Supplementary material for: Unveiling the Ongoing Expansion of Bullheads of the Genus Ameiurus (Actinopteri: Siluriformes) in the Dnipro River Basin, Ukraine: New Distributional Records and Genetic Diversity
Source: Ecol Evol. 2026 Nov 1;16(11):e74564. doi: 10.1002/ece3.74564 (PMC13724293; doi:10.1002/ece3.74564)
Supplement: Supplementary file 1 [file ECE3-16-e74564-s001.zip › Appendix.docx]

**Appendix**

**Table A1. Summary of Genetic Diversity and Neutrality Tests for *Ameiurus* Species in the Studied Populations.**

| Species | AN+AM | AM | AN | AM | AM | AM | AN | AN | AN |
| --- | --- | --- | --- | --- | --- | --- | --- | --- | --- |
| Population | all | all | all | BO | BH | KG1 | KG2 | KV | PO |
| Number of sequences used | 66 | 47 | 19 | 31 | 9 | 7 | 1 | 1 | 17 |
| Number of variable sites, S | 16 | 4 | 3 | 4 | 2 | 2 | - | - | 3 |
| Total number of mutations, Eta | 18 | 4 | 4 | 4 | 2 | 2 | - | - | 4 |
| G+C content (609.00 sites) | 0.462 | 0.462 | 0.463 | 0.462 | 0.462 | 0.462 | 0.463 | 0.463 | 0.463 |
| Number of Haplotypes, h | 10 | 5 | 5 | 4 | 4 | 3 | 1 | 1 | 5 |
| Haplotype (gene) diversity, Hd | 0.728 | 0.558 | 0.386 | 0.458 | 0.694 | 0.667 | - | - | 0.426 |
| Standard Deviation of Haplotype diversity | 0.038 | 0.064 | 0.139 | 0.089 | 0.147 | 0.16 | - | - | 0.147 |
| Nucleotide diversity (per site), Pi | 0.010 | 0.001 | 0.001 | 0.001 | 0.001 | 0.001 | - | - | 0.001 |
| Standard deviation of Pi | 0.001 | 0.000 | 0.000 | 0.000 | 0.000 | 0.000 | - | - | 0.001 |
| Average number of nucleotide differences, k | 5.892 | 0.688 | 0.678 | 0.555 | 0.889 | 0.857 | - | - | 0.750 |
| Theta (per sequence) from Eta | 3.782 | 0.906 | 1.144 | 1.001 | 0.736 | 0.816 | - | - | 1.183 |
| Theta (per site) from Eta | 0.006 | 0.001 | 0.002 | 0.002 | 0.001 | 0.001 | - | - | 0.002 |
| Neutrality Tests | | | | | | | | | |
| Tajima's D | 1.683 | -0.552 | -1.199 | -1.135 | 0.715 | 0.206 | - | - | -1.120 |
| Statistical significance | NS, P > 0.10 | NS, P > 0.10 | NS, P > 0.10 | NS, P > 0.10 | NS, P > 0.10 | NS, P > 0.10 | - | - | NS, P > 0.10 |
| Fu and Li's D* test statistic | 0.798 | -1.174 | -0.731 | -2.003 | 1.063 | -0.059 | - | - | -0.669 |
| Statistical significance | NS, P > 0.10 | NS, P > 0.10 | NS, P > 0.10 | NS, 0.10 > P > 0.05 | NS, P > 0.10 | NS, P > 0.10 | - | - | NS, P > 0.10 |
| Fu and Li's F* test statistic | 1.323 | -1.148 | -0.989 | -2.030 | 1.084 | 0 | - | - | -0.907 |
| Statistical significance | NS, P > 0.10 | NS, P > 0.10 | NS, P > 0.10 | NS, 0.10 > P > 0.05 | NS, P > 0.10 | NS, P > 0.10 | - | - | NS, P > 0.10 |
| Fu's Fs statistic | 3.667 | -1.181 | -2.203 | -0.947 | -1.283 | -0.237 | - | - | -2.088 |
| Strobeck's S statistic | 0.055 | 0.906 | 0.974 | 0.898 | 0.943 | 0.858 | - | - | 0.97 |
| P-value for Strobeck's S | 0.031 | 0.141 | 0.073 | 0.177 | 0.16 | 0.299 | - | - | 0.081 |

Note: S—number of variable sites; Eta—total number of mutations; *h*—number of haplotypes; *Hd*—haplotype diversity (with corresponding standard deviation); *Pi*—nucleotide diversity (per site, with corresponding standard deviation); *k*—average number of nucleotide differences; Theta—estimation of genetic diversity based on the number of mutations; NS—not statistically significant (P > 0.10).

**Table A2. Comparison of Morphological Traits and Identification of *A. melas* and *A. nebulosus* in Samples from New Locations.**

| Site | BE | MB | BC | A | Sp. br. | C | D | SE | ID |
| --- | --- | --- | --- | --- | --- | --- | --- | --- | --- |
| BO (n=30) | Weak nt | grey; black | grey | 17-24 | 15-21 | 19-23 | 5-7 | present, absent near tip | *A. melas* |
| BH (n=2) | weakly | black | grey | 15-18 | 16-18 | 17 | 5-6 | present, small | *A. melas* |
| KG1 (n=10) | weakly | grey; black | grey | 19-22 | 19-23 | 16-23 | 5-7 | present | *A. melas* |
| KG2 (n=2) | weakly | light | spotted | 22-23 | 14-16 | 20-22 | 5-6 | present | *A. nebulosus* |
| PO (n=17) | weakly | light grey | grey, bit spotted | 18-23 | 13-16 | 19-24 | 5-7 | well developed absent near tip;  well developed along the entire length | *A.nebulosus* |

**Note:** BE—anal fin base enlargement; MB—anal fin membrane pigmentation; BC—body colouration; A—anal fin rays total; Sp.br.—spinae branchiales (gill rakers) count; C—count of caudal fin rays; D—Dorsal-fin branched rays; SE—serrations on pectoral-fin spine; ID—identification of the species. Detailed scoring for qualitative traits is provided in the Notes to Table 4.

**Figure A1.** Matrix of pairwise genetic distances (Jukes-Cantor model) between *Ameiurus* spp. haplotypes, demonstrating species delimitation.

*Note:* Values represent genetic distances between haplotypes; colour indicates the degree of similarity/difference.

**Figure A2.** Dendrogram of *Ameiurus* population clustering from different localities, constructed using the Neighbor-Joining method on pairwise genetic distances with midpoint rooting. Differences in colours and distances between clusters reflect the level of genetic differentiation among the studied populations.

**Figure A3.** Distribution of morphological trait values for Ameiurus melas and A. nebulosus across all sampled individuals. Box plots show the distribution of meristic characters (anal fin rays, gill raker count, caudal fin rays); bar charts show the proportional distribution of qualitative characters (dorsal fin branched rays, anal fin base enlargement, anal fin membrane pigmentation, body colouration pattern, pectoral fin serration development, pectoral fin serration near tip). Trait abbreviations correspond to those defined in Table A2.
